# Collective influence of household and community capitals on agricultural employment as a measure of rural poverty in the Mahanadi Delta, India

**DOI:** 10.1007/s13280-019-01150-9

**Published:** 2019-03-09

**Authors:** Tristan Berchoux, Gary R. Watmough, Fiifi Amoako Johnson, Craig W. Hutton, Peter M. Atkinson

**Affiliations:** 1 Geography and Environmental Science, University of Southampton, University Road, Southampton, SO17 1BJ UK; 2 School of GeoSciences, University of Edinburgh, Surgeon’s Square, Drummond Street, Edinburgh, EH8 9XP UK; 3 Department of Population and Health, University of Cape Coast, Cape Coast, Ghana; 4 Lancaster Environment Centre, Lancaster University, Bailrigg, Lancaster, LA1 4YQ UK

**Keywords:** Agricultural labour, Community resources, Development economics, India, Livelihood capitals, Rural livelihoods

## Abstract

The main determinants of agricultural employment are related to households’ access to private assets and the influence of inherited social–economic stratification and power relationships. However, despite the recommendations of rural studies which have shown the importance of multilevel approaches to rural poverty, very few studies have explored quantitatively the effects of common-pool resources and household livelihood capitals on agricultural employment. Understanding the influence of access to both common-pool resources and private assets on rural livelihoods can enrich our understanding of the drivers of rural poverty in agrarian societies, which is central to achieving sustainable development pathways. Based on a participatory assessment conducted in rural communities in India, this paper differentiates two levels of livelihood capitals (household capitals and community capitals) and quantifies them using national census data and remotely sensed satellite sensor data. We characterise the effects of these two levels of livelihood capitals on precarious agricultural employment by using multilevel logistic regression. Our study brings a new perspective on livelihood studies and rural economics by demonstrating that common-pool resources and private assets do not have the same effect on agricultural livelihoods. It identifies that a lack of access to human, financial and social capitals at the household level increases the levels of precarious agricultural employment, such as daily-wage agricultural labour. Households located in communities with greater access to collective natural capital are less likely to be agricultural labourers. The statistical models also show that proximity to rural centres and access to financial infrastructures increase the likelihood of being a landless agricultural labourer. These findings suggest that investment in rural infrastructure might increase livelihood vulnerability, if not accompanied by an improvement in the provisioning of complementary rural services, such as access to rural finance, and by the implementation of agricultural tenancy laws to protect smallholders’ productive assets.

**Electronic supplementary material:**

The online version of this article (doi:10.1007/s13280-019-01150-9) contains supplementary material, which is available to authorized users.

## Introduction

Despite the Government of India’s efforts to eradicate poverty, statistics show that the percentage of farmers with land access rights has declined from 72 to 45% between 1951 and 2011 in India, whilst the percentage of landless agricultural labourers has increased from 28 to 55% (Indian Ministry of Labour and Employment 2015). This considerable rise in landless agricultural labourers is an indication of growing rural poverty (Sunam 2017). Geographically, wide variations exist both within and between rural communities, with chronic indebtedness and poverty being the highest in communities dominated by agricultural labourers. Building on the extensive literature that has looked at the political economy of agricultural employment from a caste and class perspective (e.g. Lerche 2011; Levien 2013), this research integrates a territorial approach to characterise if there are significant household and community determinants of precarious livelihoods that could enrich our understanding of the drivers of rural poverty in India. In this regard, characterising the collective influence of access to both privately owned assets and to public goods on the susceptibility of communities to landless agricultural labour could contribute to the enactment of policies targeting marginalised and vulnerable households. Incorporating local knowledge in the sustainable livelihoods framework, which has been used extensively to examine the associative relationships between access to capitals and poverty, this study examines the collective effects of access to private assets (defined as household capitals) and to common-pool resources (defined as community capitals) on the susceptibility of households to engage in precarious agricultural employment in the Mahanadi Delta. This study makes a major contribution to the literature by showing the differential impacts of private assets and common-pool resources on the dynamics of poverty and how local knowledge augments our understanding of the determinants of agricultural labour in rural India. Moreover, this research demonstrates the relevance of integrating a multilevel perspective to characterise the determinants of precarious agricultural employment, which can be replicated in different geographic settings in low- and middle-income countries.

The Mahanadi Delta in Odisha State, India is a populous delta where environmental stressors have adversely impacted livelihood opportunities, exacerbating poverty levels and driving households into chronic poverty (Chhotray and Few 2012; Dhamija and Bhide 2013). Subsistence agriculture remains the main source of employment for most of the delta’s population, with 68% of the population dependent on agriculture for their livelihoods (Registrar General and Census Commissioner 2011). The India Population and Housing Census classifies agricultural workers into cultivators and agricultural labourers. Cultivators cultivate on their own land or on land held by the Government, private persons or institutions for payment in money, kind or share. Agricultural labourers, on the other hand, work on other peoples’ land for wages and have no right of lease or contract on land. These landless agricultural labourers are amongst the most exploited and are often trapped in a vicious cycle of indebtedness and chronic poverty (Mosse et al. 2002). The problem of landless agricultural labour in the Mahanadi Delta has been compounded by high population density (623 inhabitants per square kilometre) and recurrent environmental disasters including cyclones, erosion, storm surges, floods and droughts (Bahinipati 2014), resulting in the loss of agricultural land, intensification of farming systems and persistent crop failures (Savath et al. 2014). The continual rise in landless agricultural labourers has been attributed to households’ inability to cope with the impacts of environmental shocks. Following a crop failure, agricultural households have to sell off their agricultural land to manage the immediate impacts (Hall et al. 2015). Working members of these households often become unemployed with limited livelihood opportunities to move out of poverty, either to migrate or become agricultural labourers (Williams et al. 2016). Detailed examinations of poverty structures in rural India show that households engaged in agricultural labour are amongst the poorest of the rural poor (Ravi and Engler 2015). In particular, agricultural labour is seen as a demeaning work, which provides very low wages compared to other types of daily-wage employment (Himanshu et al. 2013).

Previous research showed that employment opportunities available to rural households in low- and middle-income countries are highly dependent on access to private assets (household capitals) and on mediating factors, such as power relationships of class, caste and gender (Ellis 2000). In particular, livelihood perspectives provided a holistic approach with which to understand the systems in which rural poverty exists by considering household-level assets and capabilities, defined as livelihood capitals, which determine households’ employment opportunities. Although useful insights were provided about the factors that might influence poverty, previous studies did not fully explain the spatial disparities in terms of levels of agricultural employment that exist between communities. Community-level assets, such as access to communal natural resources (forest, lakes) and distance to services (markets, hospitals) are a significant component of rural livelihoods and poverty (Palmer-Jones and Sen 2006) and have an influence on employment opportunities at the community-level (Okwi et al. 2007). In this research, we argue that particular attention should be paid to the importance of community capitals as assets through which people are able to widen their access to resources and to economic opportunities (Lindenberg 2002; Gutierrez-Montes et al. 2009). Access to common-pool resources can contribute to households’ resilience to social, economic and environmental stresses and might influence employment opportunities by interacting with household capitals to create synergies or trade-offs (Cutter et al. 2014). Furthermore, poor management of community resources might lead to a decrease of livelihood opportunities and thus to either migration or an increase in livelihood precarity. In this regard, characterising the role of community capitals on agricultural employment could help policy-makers and practitioners to target investments at the community-level that could strengthen households’ capacities and capabilities and create employment opportunities for the poorest households. In this study, local knowledge is used to identify household and community capitals that are relevant and robust for examining the susceptibility of communities to landless agricultural labour, which is an indicator of chronic poverty.

## Conceptual framework

**Fig. 1:**
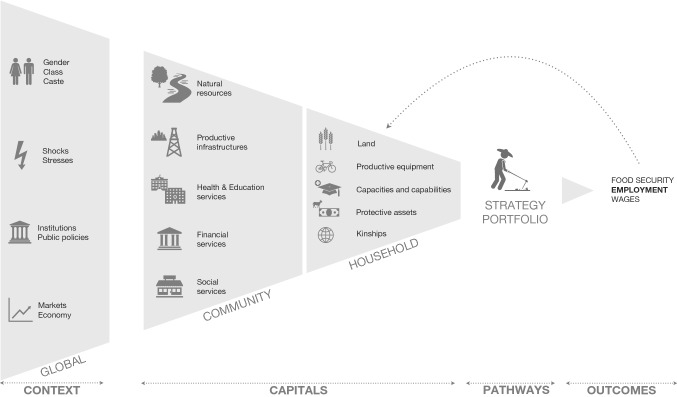
Conceptual approach underpinning the modelling of the effects of livelihood capitals on precarious livelihoods. Key examples of variables falling under each category are listed. Two levels of livelihood capitals are considered (household and community), which are shaped by the wider ecological and socio-political context. Households’ access to household and community capitals determine their choice of a set of livelihood activities, which has an influence on the outcomes they produce. Outcomes have a direct feedback effect on household capitals

Figure 1 provides the conceptual theoretical framework used in this paper. There are multiple factors that constrain or enable people’s actions (Batterbury 2008). The connections between “context” and the rest of the framework are all-encompassing. Wider structures and policies (natural context, infrastructures and systems of power) are central to the understanding of livelihoods as they modify community capitals and shape households’ access to household capitals. Investments in community capitals (through public policies) might strengthen households’ capacities and capabilities and create livelihood opportunities. On the contrary, a lack of regulation or management of community resources might lead to a decrease of livelihood opportunities and thus to either migration or an increase in livelihood precarity.

One of the main determinants of livelihood strategies that influences and conditions households’ access to resources is the socio-economic group to which its members belong, defined by gender, age, wealth, ethnicity, class and caste. These factors play a major role in the household’s power relationships and social networks by removing (or creating) barriers to their use of livelihood assets. The socioeconomic hierarchy conceptualised by gender, class and caste is linked to ownership and income and plays a significant dimension in access to assets and to the type of activities conducted by people. Disadvantaged caste members can suffer from social and economic exclusion, women can suffer from a lack of access to certain types of assets or from a social unacceptability to undertake some activities, and age will have an influence on the members’ employment opportunities. Moreover, high status employment is dominated by upper caste, while physical labour and low status jobs are mostly performed by lower caste or *dalit*. As a consequence, income disparity, employment opportunities and access to capitals are highly associated with the systems of power, and especially with the caste system.

Capitals are resources that people have access to, which can be private goods (household capitals) or public goods (community capitals). Household capitals are grouped into a set of five categories: natural (natural resource stocks), physical (productive assets), financial (liquidities and protective assets), human (capabilities and capacities of the households) and social (networks and kinships). Similarly, five categories of community capitals can be differentiated (Flora et al. 2015): natural (common resources), financial (availability of financial amenities), physical (availability of productive infrastructures, such as road networks, markets and industries), human (availability of schools and hospitals) and social (social balance within a community and availability of social infrastructures). Based on their access to community and household assets, households put in place a range of livelihood activities to achieve their basic needs. Employment opportunities are influenced by one’s access to household and community capitals and is one of the main outcomes pursued by households (Fenichel et al. 2016).

## Materials and methods

**Fig. 2:**
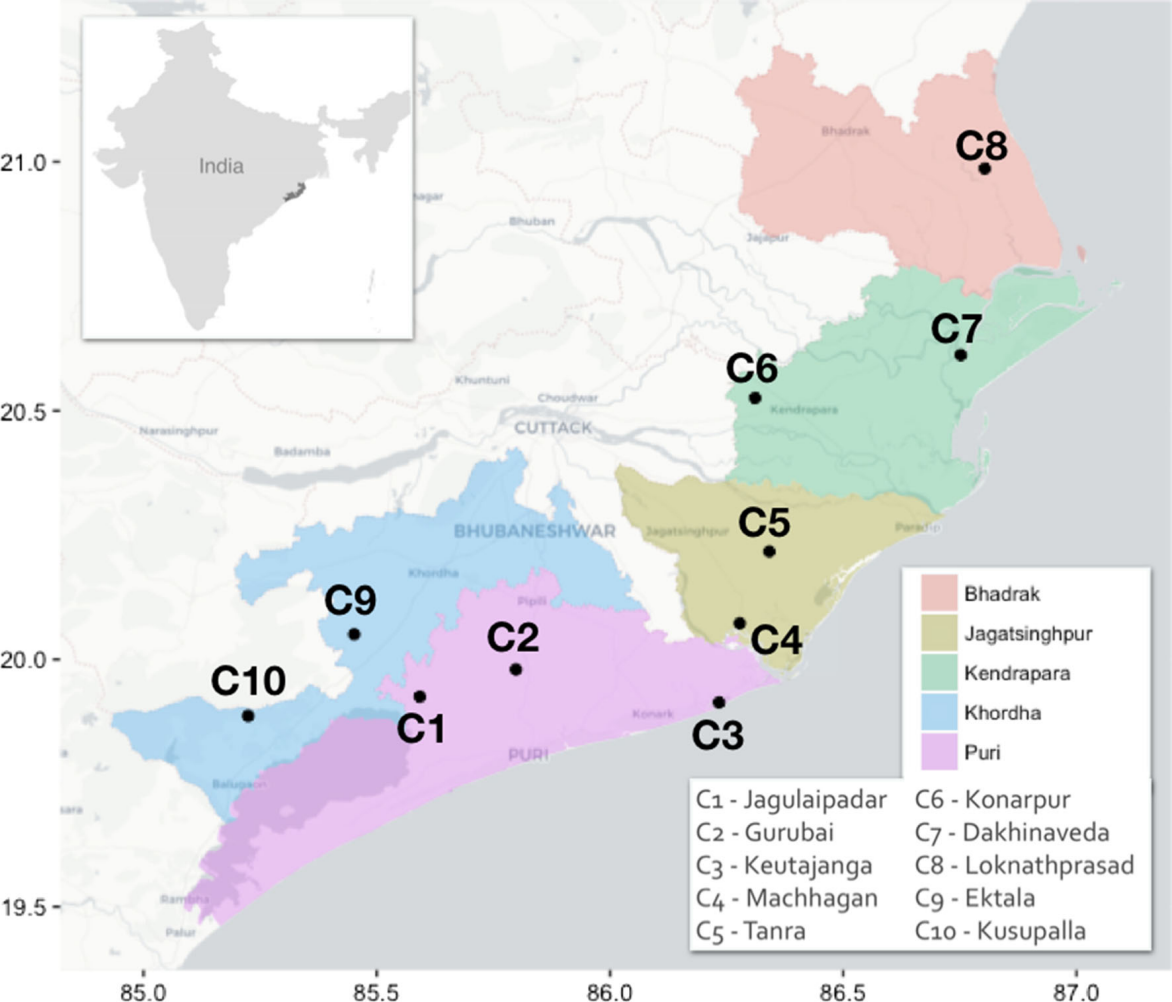
Location of the sampled communities across the Mahanadi Delta in India. Rapid rural appraisals were conducted in ten communities (C1–C10), selected according to their level of vulnerability, their location and the dominant land cover

The study focused on the Mahanadi Delta located within the state of Odisha in East India (Fig. 2). The study area covered all five districts located within the Mahanadi river delta: Bhadrak, Jagatsinghpur, Kendrapara, Khorda and Puri.

**Fig. 3:**
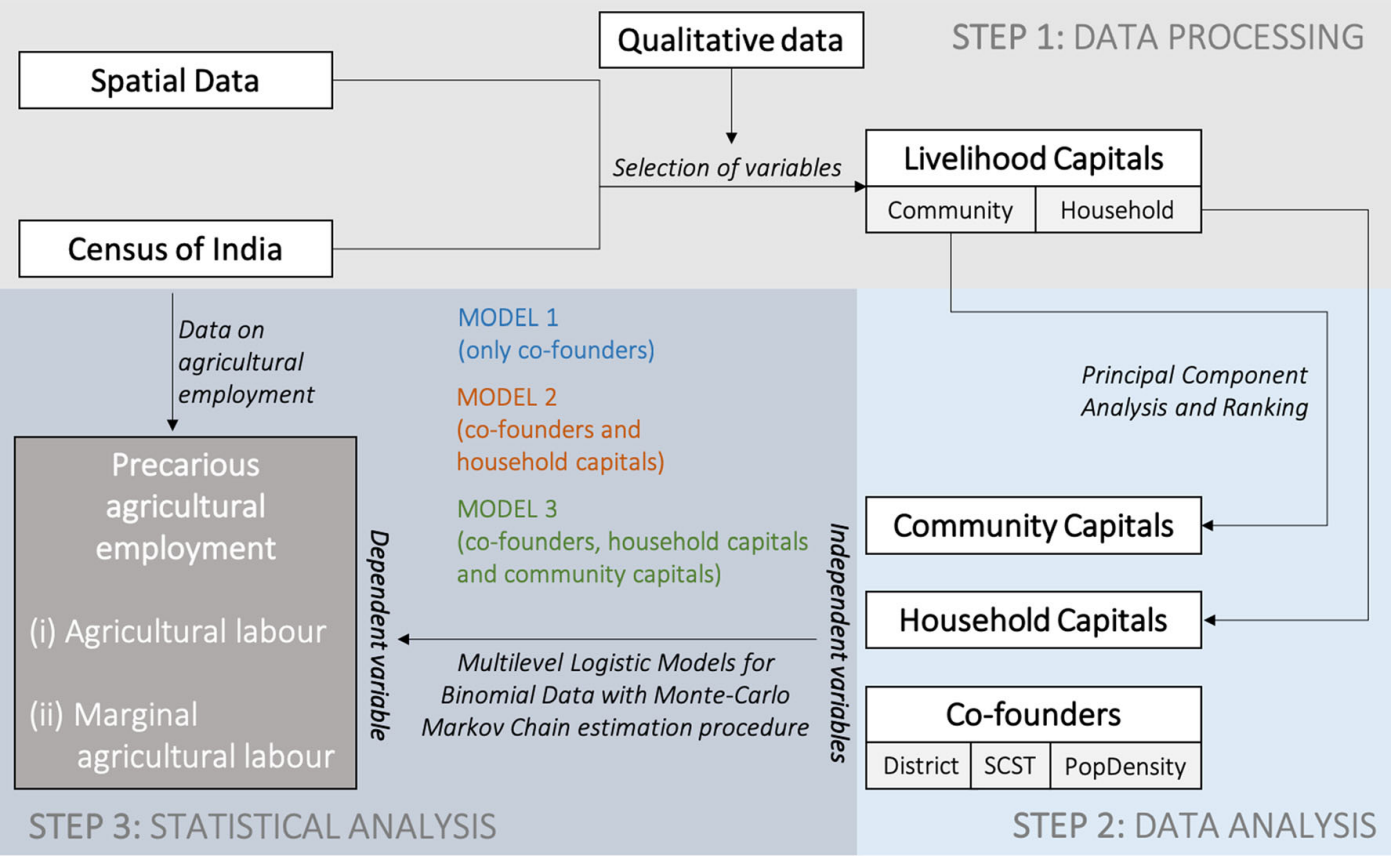
Study methodology. Flowchart describing the study methodology in three major steps: (i) data processing, (ii) data analysis and (iii) statistical analysis

Figure 3 shows a schematic diagram of the methodology with the three major steps followed in this research: (i) data processing; (ii) data analysis; and (iii) statistical analysis.

### Data processing

Fieldwork was conducted between February and May 2016 to identify indicators that stakeholders, experts and local residents perceive as representative and robust to examine the effects of each capital on their livelihoods. A Rapid Rural Appraisal (RRA) was used as the principal method for data collection to highlight the perceptions and opinions of communities (see Supplementary Material S1). This method enables local people to share their knowledge, and discuss and analyse their situation using their own terms (Mukherjee 2005). In total, ten villages were sampled to represent a variety of cases based on their socio-economic characteristics and on the main livelihood activities conducted by households (Fig. 2). Different activities were used to cross-check the data acquired and to cover all aspects of livelihood systems. First, a participatory workshop was held as a focus group and general information about the village and the evolution of its infrastructure was discussed. Differences within the community regarding livelihood assets and strategies were investigated. Once the different categories were identified by the participants, they quantified the proportion of households falling into each category. The last activity was a participatory photography workshop using the *photovoice* methodology (Wang and Burris 1997) on the theme of “Key assets to achieve your livelihoods”; a theme broad enough to let the participants themselves highlight the different roles that community and household capitals play in their decision to pursue an economic activity.

Based on the RRA, we selected data to measure livelihood capitals, including demographic, infrastructure, amenities, and environmental indicators. The data used for the analysis were derived from the 2011 India Population and Housing Census, Open Street Map data (OSM) and 2011 Bhuvan^1^ satellite imagery. The Census and remotely sensed satellite sensor data were adopted because they provide detailed data at a finer spatial resolution (community) and are publicly accessible online. The demographic, infrastructure and amenities data used in the analysis were derived from the 2011 India Population and Housing Census (Registrar General and Census Commissioner 2011). The census indicators comprise population enumeration including cultivators (marginal and main), agricultural labourers and entrepreneurs (marginal and main), education, literacy, mean income and expenditure, access to health facilities, drinking water, communication, banking, recreational and cultural facilities, power supply and natural resources. Only one economic activity is recorded per person and is classified as main (work for more than 6 months) or marginal (work for less than 6 months). The use of environmental data has a relatively long tradition within rural development studies due to the fact that rural livelihoods and land use are intertwined (Behera et al. 2016). The Geographic Information System software QGIS was used to extract different environmental indicators at the community level and also to compute travel times to closest resources. Our calculations cover an area extending 100 km beyond the administrative boundary of the study area to avoid edge effects. The main features extracted from the Bhuvan land cover dataset (25 m resolution for 2011) were built-up area, forest cover (evergreen/deciduous/shrubs/mangroves), agricultural land (cropland/plantation/fallow) and waterbodies.

### Data analysis

Based on the findings from the RRA and on data quality and availability, a multidimensional matrix of indicators was identified to quantify each of the household and community capitals. Given the high correlation amongst the selected variables, a principal component analysis was used to circumvent the problem of multicollinearity and to derive a single factor score for each capital. Multiple factors were not combined as this would have distorted what the component represents and would have made it difficult to interpret (McKenzie 2005). After ensuring that the factor loadings corresponded with the conceptualisation of each capital based on the RRA exercise, the first factor score was selected to represent each capital and categorised into quintiles to show the communities with least access to each capital and those with the highest access (see Supplementary Material S2). Thirteen spatially explicit variables were used to represent the five household capitals (Table 1) and fourteen spatially explicit variables were used to represent the five community capitals (Table 2).

**Table 1:** List of variables used for the quantification of household livelihood capitals. The associated factor loading retrieved from the PCA represents the weight of each variable in the construction of each livelihood capital. *Source* Census

Category	Variables	Weight	Justification from Rapid Rural Appraisal
Natural capital			
Cropland	Average area sown per cultivator	0.382	Influences households’ incomes and food security
Tree plantation	Average area of tree crops per cultivator	0.398	Enables households to generate extra incomes
Pasture	Average area of pasture per cultivator	0.440	Enables households to develop livestock rearing
Physical capital			
Electricity	No access to electricity (%)	− 0.083	Lack of electricity prevents households to conduct their livelihood activity (to operate agricultural pumps and machinery)
Means of transportation	Access to bicycle (%)	0.445	Enables households to look for new outlets for their production and increase their access to nearby social services through the reduction of travel times
	Access to motorcycle (%)	0.530	
	Access to car (%)	0.400	
Human capital			
Dependency ratio	Number of inactive per active person	− 0.687	High dependency limits the range of activities that the household can put in place and reduces investment
Illiteracy	Illiterate individuals (%)	− 0.687	Educated members were a strength for one household because they “did not suffer from unemployment”
Financial capital			
Financial services	Access to financial services (%)	0.682	Enables households to invest in their other capitals and develop their livelihood opportunities
Housing conditions	“Dilapidated” houses (%)	− 0.682	Value and condition of housing represents the financial condition of households
Social capital			
Marital status	No married couples (%)	− 0.395	Marriage is one of the most important kinship encountered at the household level in rural settings
Mobile phone	Ownership of mobile phone (%)	0.569	Mobile phones enable households to communicate with migrants and strengthen networks

**Table 2:** List of variables used for the quantification of community livelihood capitals. The associated factor loading retrieved from the PCA represents the weight of each variable in the construction of each livelihood capital

Category	Variables	Source	Weight	Justification from Rapid Rural Appraisal
Natural capital				
Cropland	Total cropland area	Bhuvan	0.650	Greater amount of land in the community increases opportunities for agricultural livelihoods
Forest	Total area of forest in the community	Bhuvan	0.198	Access to forest can provide extra income, food and energy supply
Open-water	Travel time to aquaculture areas	OSM	− 0.589	Access to open-water resources can provide extra income and food supply
Irrigation	Proportion of cropland with irrigation	Census	0.343	Public irrigation infrastructures enable farmers to grow multiple crops a year
Physical capital				
Markets	Travel time to closest market	Census	− 0.534	Proximity to markets enable farmers to sell their products and to look for alternative livelihoods
Industry	Travel time to closest industrial zone	OSM	− 0.534	Proximity to industrial areas increases households’ opportunities for alternative livelihoods
Human capital				
Health facilities	Travel time to closest hospital	Census	− 0.704	Proximity to hospitals enables households to cope more rapidly with shocks on their labour force
Schools	Travel time to closest secondary school	Census	− 0.704	Proximity to schools increases the capacity of youth members of the household
Financial capital				
Banks	Travel time to closest bank	Census	− 0.582	Proximity to banks enables households to get financial services and access to national poverty schemes
ATM	Travel time to closest ATM	Census	− 0.408	ATMs enable households to get access to cash and was seen as important for livelihood opportunities
Public Distribution System	Travel time to closest PDS centre	Census	− 0.689	Proximity to PDS enables the poorest households to get access to national poverty schemes
Social capital				
Community centre	Travel time to closest community centre	Census	− 0.341	Community centres are key amenities for socialisation in rural areas
Recreation	Travel time to closest sport field	Census	− 0.677	Recreational infrastructures prevent youth to migrate and is a lever to find livelihood opportunities
Union	Travel time to closest Self-Help Group	Census	− 0.319	Self-Help Groups are powerful networking institutions that can provide livelihood opportunities

### Statistical analysis

Multilevel logistic regression was used to investigate the effects of household and community capitals on the odds of working as an agricultural labourer. Two response variables were considered: (i) agricultural labourers, derived as the ratio of agricultural labourers to total population engaged in agricultural activities; and (ii) marginal agricultural labourers, computed as the ratio of agricultural labourers who work less than 6 months per year to the total population engaged in agricultural labour. The proportions of the response variables of interest varied continuously over the range of 0 and 1. Thus, fitting a linear regression model to this data risked predicting invalid values outside the range of 0 and 1. In this regard, a Generalised Linear Model (GLM) with a logit link was adopted, specifying the total number of adults who were engaged in (i) agricultural activities or (ii) agricultural labour as the denominator, to ensure that predicted values remained in the range of 0 and 1. Contextual factors, such as socio-political and ecological contexts, strongly impact employment opportunities, outcomes and the ability of households to implement coping strategies (Cinner et al. 2018). Multilevel logistic regression was used to control for such factors, by allowing the model to vary at the Tehsil level (administrative division level 3 earmarked for administration and development in India). Three-level GLM models were fitted with 3,620 rural communities (level 1) nested in 2420 Gram Panchayat (level 2), further nested in 67 Tehsils (level 3).

A sequential model-building process was used to examine the extent to which the household and community capitals explain the odds of working as an agricultural labourer, accounting for important confounders: districts to which the communities belong (District), population density of the communities (PopDensity) and proportion of scheduled castes and tribes (SCST). For each response variable, three models were fitted using MLwiN 3.01 (Charlton et al. 2017). Model 1 accounted for the confounders and random effects:

1 $$\text{logit}(\pi_{ijk}) = \log \left( \frac{\pi_{ijk}}{1-\pi_{ijk}}\right) = \beta_{ojk} + \beta_{1} \text{District}_{ijk} + \beta_{2} \text{PopDensity}_{ijk} + \beta_{3} \text{SCST}_{ijk},$$

Model 2 added the household capitals (HC) to the confounders and random effects:

2 $$\text{logit}(\pi_{ijk}) = \log \left( \frac{\pi_{ijk}}{1-\pi_{ijk}}\right) = \beta_{ojk} + \beta_{1} \text{District}_{ijk} + \beta_{2} \text{PopDensity}_{ijk} + \beta_{3} \text{SCST}_{ijk} + \beta_{4} \text{Nat}\_\text{HC}_{ijk} + \beta_{5} \text{Phy}\_\text{HC}_{ijk} + \beta_{6} \text{Hum}\_\text{HC}_{ijk} + \beta_{7} \text{Fin}\_\text{HC}_{ijk} + \beta_{8} \text{Soc}\_\text{HC}_{ijk},$$

whilst Model 3 further added the community capitals (CC) to the household capitals, confounders and random effects:

3 $$\text{logit}(\pi_{ijk}) = \log \left( \frac{\pi_{ijk}}{1-\pi_{ijk}}\right) = \beta_{ojk} + \beta_{1} \text{District}_{ijk} + \beta_{2} \text{PopDensity}_{ijk} + \beta_{3} \text{SCST}_{ijk} + \beta_{4} \text{Nat}\_\text{HC}_{ijk} + \beta_{5} \text{Phy}\_\text{HC}_{ijk} + \beta_{6} \text{Hum}\_\text{HC}_{ijk} + \beta_{7} \text{Fin}\_\text{HC}_{ijk} + \beta_{8} \text{Soc}\_\text{HC}_{ijk} + \beta_{9} \text{Nat}\_\text{CC}_{ijk} + \beta_{10} \text{Phy}\_\text{CC}_{ijk} + \beta_{11} \text{Hum}\_\text{CC}_{ijk} + \beta_{12} \text{Fin}\_\text{CC}_{ijk} + \beta_{13} \text{Soc}\_\text{CC}_{ijk},$$

where $\pi_{ijk}$ refers to the probability (i) of working as an agricultural labourer and (ii) of working as a marginal agricultural labourer for the community *i* in the *Tehsilj* and Gram Panchayat *k*. The random effect $\beta_{0j}$ is defined as the sum of the intercept $\beta_0$ and a random effect varying at the Tehsil level $U_{oj}$. As the response variable is binomial, we used a linearisation method in the model to transform the discrete response model (binomial) to a continuous response model (Goldstein 2003), with a Bayesian modelling approximation method to estimate the unknown parameters of interest in the model. This approach used a combination of two Monte Carlo Markov Chain (MCMC) procedures, Gibbs sampling and Metropolis-Hastings sampling, to generate a large number of simulated random draws from the joint posterior distribution of all the parameters. It then used these random draws to form a univariate summary of the underlying distributions, which is useful for producing accurate interval estimates.

### Research limitations

The approach of this research was to scale up to a larger spatial extent the knowledge co-created with the participants of rapid rural appraisals in order to characterise how drivers of precarious livelihoods vary locally, due to their access to community capitals. The study used proxies to characterise livelihood capitals to quantify the diversity of factors identified by participants in order to characterise their effect on precarious agricultural employment. However, indicators of livelihood capitals have been criticised, as they simplify the complexity of households’ assets and capabilities to an aggregated number, which may lead to fallacious conclusions. The manual binning of variables under certain capitals is subject to interpretation, and its relevance and reproducibility might be questionable. Furthermore, due to the date mismatch between our fieldwork (2016) and the collection of the quantitative data (2011), there is also potential for bias in the selection of the variables used for the quantification of livelihood capitals. Since the aim of the study required access to publicly available data at the village level, it was not possible to use another dataset than the Census, the most recent Census of India being 2011 at the time of conducting this research. To ensure consistency in our statistical modelling, we thus decided to also use remote sensing data from 2011. An issue that was not addressed in this study was whether the perception of livelihood capitals by participants was different between 2011 and 2016. Finally, access to livelihood capitals is controlled by overarching systems of power (defined by class, caste and gender), which have been shown to be one of the main causal determinants of poverty in India (Lerche 2009). Therefore, this research avoided inferring any definite causal relationships throughout because of uncertainties surrounding the effects of livelihood capitals on precarious agricultural employment. The links between context, livelihood capitals and agricultural employment are complex, and the list of potential interactions and mediating factors is vast and often unquantifiable. It was not in the scope of this research to provide an in-depth understanding of the role of such factors. Instead this research focused on exploring how large datasets could be used in combination with participatory knowledge to characterise existing effects, acknowledging and accounting for the fact that they are context and place dependent. In spite of the above limitations, this research adds to our understanding of the determinants of precarious agricultural employment by providing an approach that can enable researchers, policy-makers and practitioners to investigate the effects of common-pool resources on rural development.

## Results

### Multilevel logistic model for agricultural labour

Three different models were fitted to analyse the effects of the different explanatory variables on agricultural labour (Table 3). The lowest Akaike information criterion (AIC) was obtained when both community and household capitals were included in the model (Model 3, AIC decreased by 16,354 compared to Model 1 and by 1,105 compared to Model 2). The large decline in the AIC showed that both types of capitals were required in the model, thus, indicating that Model 3 explained the most variation in the independent variable.

**Table 3:** Results of the multilevel logistic models for the proportion of the agricultural workers who were labourers. The dependent variable represented the proportion of workers engaged in agriculture who were working as agricultural labourers. Model 1 was the null model in which only the confounders were considered. Model 2 tested the effect of household capitals. Model 3 took the two levels of livelihood capitals into account

Background characteristics and capitals	Model 1	Model 2	Model 3
	OR [95% CI]	OR [95% CI]	OR [95% CI]
Confounders			
District			
Puri	1.00	1.00	1.00
Khordha	1.37 [1.02, 1.85]*	1.36 [1.16, 1.61]***	1.27 [1.08, 1.51]**
Jagatsinghpur	0.86 [0.74, 0.99]*	1.48 [1.13, 1.95]**	1.43 [1.15, 1.79]**
Bhadrak	0.78 [0.66, 0.93]**	1.13 [0.87, 1.48]	1.05 [0.86, 1.30]
Kendrapara	0.71 [0.57, 0.88]**	0.95 [0.75, 1.20]	1.05 [1.10, 1.30]
Population density	1.02 [0.99, 1.05]	0.53 [0.51, 0.55]***	0.58 [0.56, 0.60]***
Castes and tribes	5.39 [5.10, 5.69]***	3.87 [3.67, 4.07]***	3.66 [3.44, 3.89]***
Household capitals			
Natural			
Very high		1.00	1.00
High		0.39 [0.38, 0.41]***	0.39 [0.37, 0.40]***
Moderate		0.30 [0.29, 0.31]***	0.29 [0.28, 0.30]***
Low		0.20 [0.19, 0.20]***	0.19 [0.18, 0.19]***
Very low		0.11 [0.11, 0.12]***	0.11 [0.11, 0.12]***
Physical			
Very high		1.00	1.00
High		1.15 [1.12, 1.18]***	1.15 [1.11, 1.19]***
Moderate		1.16 [1.13, 1.20]***	1.18 [1.15, 1.22]***
Low		1.17 [1.13, 1.20]***	1.20 [1.16, 1.24]***
Very low		1.24 [1.20, 1.28]***	1.28 [1.23, 1.33]***
Human			
Very high		1.00	1.00
High		1.49 [1.44, 1.55]***	1.52 [1.46, 1.58]***
Moderate		1.24 [1.20, 1.28]***	1.24 [1.20, 1.29]***
Low		1.18 [1.14, 1.22]***	1.17 [1.13, 1.21]***
Very low		1.18 [1.15, 1.22]***	1.17 [1.13, 1.20]***
Financial			
Very high		1.00	1.00
High		1.04 [1.01, 1.07]**	1.01 [0.98, 1.04]
Moderate		1.05 [1.02, 1.08]**	1.01 [0.98, 1.04]
Low		1.23 [1.19, 1.27]***	1.22 [1.18, 1.26]***
Very low		1.27 [1.22, 1.31]***	1.22 [1.18, 1.27]***
Social			
Very high		1.00	1.00
High		1.16 [1.13, 1.20]***	1.11 [1.08, 1.15]***
Moderate		1.16 [1.13, 1.20]***	1.11 [1.07, 1.15]***
Low		1.22 [1.18, 1.26]***	1.12 [1.08, 1.17]***
Very low		1.25 [1.20, 1.29]***	1.16 [1.12, 1.19]***
Community capitals			
Natural			
Very high			1.00
High			1.07 [1.04, 1.11]***
Moderate			1.08 [1.04, 1.12]***
Low			1.22 [1.18, 1.26]***
Very low			1.25 [1.20, 1.29]***
Physical			
Very high			1.00
High			0.98 [0.95, 1.01]
Moderate			1.01 [0.98, 1.04]
Low			1.09 [1.05, 1.13]***
Very low			1.12 [1.09, 1.16]***
Human			
Very high			1.00
High			1.00 [0.97, 1.03]
Moderate			1.01 [0.98, 1.04]
Low			1.04 [1.01, 1.07]*
Very low			1.15 [1.12, 1.19]***
Financial			
Very high			1.00
High			0.94 [0.92, 0.97]***
Moderate			0.91 [0.88, 0.94]***
Low			0.88 [0.86, 0.91]***
Very low			0.76 [0.73, 0.79]***
Social			
Very high			1.00
High			0.92 [0.90, 0.94]***
Moderate			0.91 [0.88, 0.94]***
Low			0.81 [0.79, 0.84]***
Very low			0.80 [0.77, 0.83]***
Random effects			
Tehsil	1.16 [1.08, 1.24]***	1.13 [1.06, 1.19]***	1.14 [1.07, 1.21]***
Gram	2.97 [2.73, 3.24]***	2.21 [2.08, 2.36]***	2.21 [2.07, 2.35]***
Intersect	0.56 [0.52, 0.62]***	1.18 [1.09, 1.28]***	1.29 [1.16, 1.43]***

Significance level: *** *p* < 0.001, ** *p* < 0.01, * *p* < 0.05

Model 3 showed that communities located in the Districts Khordha and Jagatsinghpur had higher odds of working as an agricultural labourer compared to those in Puri ($\hbox{OR}_{\text{Khordha}} = 1.27$, 95% CI 1.08, 1.51; $\hbox{OR}_{\text{Jagatsinghpur}} =1.43$, 95% CI 1.15, 1.79). There was also a significant negative effect of population density on the odds of working as an agricultural labourer (OR = 0.58, 95% CI 0.56, 0.60). Moreover, belonging to disadvantaged groups (scheduled castes and tribes) increased the odds of working as an agricultural labourer (OR = 3.66, 95% CI 3.44, 3.89).

Concerning the effects of household capitals and agricultural labour, the results obtained from Model 3 showed that the five capitals had a statistically significant effect on the odds of working as an agricultural labourer. Agricultural households with very low access to human capital were more likely to be agricultural labourers compared to those with very high human capital ($\hbox{OR}_{\text{Very Low}} = 1.52$, 95% CI 1.46, 1.58). It was also apparent that a lower access to financial ($\hbox{OR}_{\text{Very Low}} = 1.22$, 95% CI 1.18, 1.27) and social capital ($\hbox{OR}_{\text{Very Low}} = 1.16$, 95% CI 1.12, 1.19) increased the odds of working as an agricultural labourer. The odds of working as an agricultural labourer were also significantly higher for households with very low household physical capital ($\hbox{OR}_{\text{Very Low}} = 1.28$, 95% CI 1.23, 1.33) compared to households with very high household physical capital. Regarding household natural capital, a very low ($\hbox{OR}_{\text{Very Low}} = 0.11$, 95% CI 0.11, 0.12) access to this capital decreased the odds of engaging in agricultural labour compared to households with very high household natural capital.

As Table 3 shows, community natural, physical and human capitals had significant effects on the odds of working as an agricultural labourer. Actually, households with a very low access to community natural ($\hbox{OR}_{\text{Very Low}} = 1.25$, 95% CI 1.20, 1.29), physical ($\hbox{OR}_{\text{Very Low}} = 1.12$, 95% CI 1.09, 1.16) or human ($\hbox{OR}_{\text{Very Low}} = 1.15$, 95% CI 1.12, 1.19) had higher odds of working as an agricultural labourer than households with a very high access to them. On the contrary, the odds of working as an agricultural labourer decreased with lower access to community financial capital ($\hbox{OR}_{\text{Very Low}} = 0.76$, 95% CI 0.73, 0.79). Similarly, the odds of working as an agricultural labourer decreased for households with lower community social capital ($\hbox{OR}_{\text{Very Low}} = 0.80$, 95% CI 0.77, 0.83).

### Multilevel logistic model for marginal agricultural labour

Three models were fitted to analyse the effects of community and household livelihood capitals on the odds of working for less than 6 months (marginal activity) for agricultural labourers. The results obtained from the different models are summarised in Table 4. The lowest AIC was obtained by adding both household and community capitals to the model (Model 3, AIC decreased by 4712 compared to Model 1 and by 595 compared to Model 2), indicating that Model 3 explained the most variation in the independent variable.

**Table 4:** Results of the multilevel logistic models for the proportion of agricultural labourers who were employed marginally. The dependent variable represents the proportion of agricultural labourers who were working for less than 6 months per year. Model 1 was the null model in which only the confounders were considered. Model 2 tested the effect of household capitals. Model 3 took the two levels of livelihood capitals into account

Background characteristics and capitals	Model 1 OR [95% CI]	Model 2 OR [95% CI]	Model 3 OR [95% CI]
Confounders			
District			
Puri	1.00	1.00	1.00
Khordha	1.09 [0.88, 1.37]	0.99 [0.81, 1.21]	0.98 [0.81, 1.19]
Jagatsinghpur	1.09 [0.88, 1.35]	1.02 [0.85, 1.23]	1.08 [0.84, 1.40]
Bhadrak	1.08 [0.95, 1.23]	1.21 [0.95, 1.55]	1.17 [0.99, 1.38]
Kendrapara	1.06 [0.89, 1.26]	1.05 [0.90, 1.24]	1.09 [1.10, 1.39]
Population density	1.25 [1.21, 1.30]***	0.97 [0.94, 1.01]	0.98 [0.95, 1.02]
Castes and tribes	3.26 [3.04, 3.50]***	3.10 [2.92, 3.30]***	2.87 [2.68, 3.08]***
Household capitals			
Natural			
Very high		1.00	1.00
High		0.61 [0.59, 0.63]***	0.63 [0.61, 0.65]***
Moderate		0.53 [0.51, 0.55]***	0.54 [0.53, 0.56]***
Low		0.42 [0.40, 0.43]***	0.42 [0.41, 0.44]***
Very low		0.35 [0.34, 0.37]***	0.36 [0.35, 0.38]***
Physical			
Very high		1.00	1.00
High		1.00 [0.96, 1.03]	1.00 [0.96, 1.05]
Moderate		1.00 [0.96, 1.04]	1.02 [0.98, 1.07]
Low		1.17 [1.12, 1.22]***	1.18 [1.13, 1.24]***
Very low		1.29 [1.23, 1.34]***	1.33 [1.26, 1.40]***
Human			
Very high		1.00	1.00
High		1.14 [1.10, 1.18]***	1.12 [1.07, 1.17]***
Moderate		1.27 [1.22, 1.32]***	1.21 [1.17, 1.26]***
Low		1.45 [1.39, 1.51]***	1.42 [1.36, 1.48]***
Very low		2.06 [1.97, 2.16]***	1.99 [1.91, 2.09]***
Financial			
Very high		1.00	1.00
High		0.98 [0.94, 1.02]	1.02 [0.97, 1.07]
Moderate		1.08 [1.04, 1.12]***	1.10 [1.05, 1.15]***
Low		1.08 [1.03, 1.13]***	1.11 [1.05, 1.16]***
Very low		1.18 [1.13, 1.24]***	1.22 [1.16, 1.28]***
Social			
Very high		1.00	1.00
High		1.03 [0.99, 1.07]	1.03 [0.99, 1.07]
Moderate		1.02 [0.98, 1.07]	1.00 [0.96, 1.05]
Low		0.99 [0.95, 1.03]	0.96 [0.92, 1.01]
Very low		0.85 [0.82, 0.89]***	0.85 [0.81, 0.89]***
Community capitals			
Natural			
Very high			1.00
High			1.00 [0.96, 1.04]
Moderate			0.98 [0.94, 1.03]
Low			0.85 [0.82, 0.89]***
Very low			0.83 [0.79, 0.86]***
Physical			
Very high			1.00
High			1.00 [0.95, 1.04]
Moderate			0.98 [0.94, 1.03]
Low			0.97 [0.93, 1.01]
Very low			0.90 [0.87, 0.94]***
Human			
Very high			1.00
High			1.10 [1.06, 1.14]***
Moderate			1.11 [1.08, 1.15]***
Low			1.14 [1.09, 1.18]***
Very low			1.16 [1.12, 1.20]***
Financial			
Very high			1.00
High			1.03 [0.99, 1.08]
Moderate			1.07 [1.03, 1.11]***
Low			1.17 [1.13, 1.23]***
Very low			1.20 [1.16, 1.26]***
Social			
Very high			1.00
High			0.95 [0.91, 0.99]*
Moderate			0.92 [0.90, 0.95]***
Low			0.82 [0.79, 0.85]***
Very low			0.73 [0.70, 0.77]***
Random effects			
Tehsil	1.05 [1.00, 1.10]*	1.04 [1.01, 1.08]**	1.05 [1.01, 1.09]**
Gram	5.94 [5.12, 6.90]***	5.32 [4.62, 6.12]***	5.40 [4.69, 6.22]***
Intersect	0.17 [0.16, 0.19]***	0.21 [0.19, 0.25]***	0.22 [0.19, 0.26]***

Significance level: *** *p* < 0.001, ** *p* < 0.01, * *p* < 0.05

It was apparent from Model 3 that the likelihood of having a marginal activity for agricultural labourers was not influenced by the district in which households were located. Similarly, the model showed that population density did not have a significant effect on the odds of working as a marginal agricultural labourer. On the contrary, people belonging to disadvantaged groups (scheduled castes and tribes) had higher odds of working for less than 6 months per year (OR 2.87, 95% CI 2.68, 3.08).

Agricultural labourers who had a very low access to household physical ($\hbox{OR}_{\text{Very Low}} = 1.33$, 95% CI 1.26, 1.40), human ($\hbox{OR}_{\text{Very Low}} = 1.99$, 95% CI 1.91, 2.09) or financial ($\hbox{OR}_{\text{Very Low}} = 1.22$, 95% CI 1.16, 1.28) capital had greater odds of having a marginal activity compared to agricultural labourers with a very high access to these capitals. On the contrary, odds of having a marginal activity increased when agricultural labourers had a lower access to household natural capital ($\hbox{OR}_{\text{Very Low}} = 0.36$, 95% CI 0.35, 0.38) or to household social capital ($\hbox{OR}_{\text{Very Low}} = 0.85$, 95% CI 0.81, 0.89).

Amongst community capitals, the model showed that agricultural labourers who had a very low access to community natural ($\hbox{OR}_{\text{Very Low}} = 0.83$, 95% CI 0.79, 0.86), physical ($\hbox{OR}_{\text{Very Low}} = 0.90$, 95% CI 0.87, 0.94) or social ($\hbox{OR}_{\text{Very Low}} = 0.73$, 95% CI 0.70, 0.77) capital were less likely to be employed for less than 6 months. However, a very low access to community human ($\hbox{OR}_{\text{Very Low}} = 1.16$, 95% CI 1.12, 1.20) or financial ($\hbox{OR}_{\text{Very Low}} = 1.20$,  95% CI 1.16, 1.26) capitals increased the odds of working as a marginal agricultural labourer.

## Discussion

This research provides an innovative empirical development to livelihood studies by combining census data with satellite remote sensing products to explore the collective influence of household and community capitals on agricultural employment. More specifically, the initial objective of this investigation was to demonstrate the extent to which both household and community capitals play a significant role in the likelihood of being a landless agricultural labourer, an effect that has not yet been investigated. This study shows that community resources and household capitals should be considered separately as they do not necessarily have the same effects on the likelihood of being a landless agricultural labourer.

Rural India’s most vulnerable households are daily-wage agricultural labourers and those who only have a marginal activity are considered as the poorest of the poor (Pattenden 2010). Engaging in such livelihoods is a source of distress for households, which drives migration and reinforces rural poverty (Wang et al. 2011). The combination of the findings emerging from this research shows that working as an agricultural labourer is influenced by access to household capitals, which is consistent with previous research in the field of livelihood studies. The current study brings a new perspective on these effects by demonstrating that community capitals also have an influence on households’ livelihood opportunities. A summary of the influence of both household and community capitals on agricultural labour is presented in Table 5.

**Table 5:** Likelihood to engage in agricultural labour. The results show the likelihood to engage in agricultural labour for agricultural households (left) and the likelihood to only have a marginal activity for agricultural labourers (right). The results presented here are derived from the models including both community and household livelihood capitals. Arrows represent the direction of significant effects

Livelihood capitals		Agricultural livelihood activities	
Type	Level	Agricultural labourer (compared to cultivator)	Marginal agricultural labourer (compared to main)
Natural	*Household*	↑	↑
	*Community*	↓	↑
Physical	*Household*	↓	↓
	*Community*	↓	↑
Human	*Household*	↓	↓
	*Community*	↓	↓
Financial	*Household*	↓	↓
	*Community*	↓	↓
Social	*Household*	↓	↓
	*Community*	↑	↑

### Livelihood capitals and agricultural labour

#### Natural capital

The study showed significant effects of household natural capital on levels of agricultural labour. Communities with larger farms (including cropland, tree plantation and pasture) are more likely to have a larger proportion of households engaging in agricultural labour, and especially in marginal agricultural labour. This result confirms the findings of Manjunatha et al. (2013) who demonstrated that households are more likely to engage in precarious forms of employment when they are located in communities where natural resources are only owned by few large-scale farmers. Smallholders sell their land to larger farm holders due to an inability to cope with recurrent crop failures, driving them into agricultural labour (Levien 2013). On the other hand, the findings show that households located in communities with a greater access to community natural capital are less likely to be agricultural labourers. This finding provides further support to the hypothesis that greater access to common-pool natural resources enables more households to engage in cultivation (de Sherbinin et al. 2008). However, the results also show that agricultural labourers are more likely to have a marginal activity when they are located in a village with a larger community natural capital. This finding supports the hypothesis that communities with access to irrigation facilities require less labour throughout the year compared to rainfed agricultural systems.

#### Physical capital

We found that access to means of transportation and to electricity had a negative effect on agricultural labour. This finding corroborates the results from the Rapid Rural Appraisals, which showed that electricity allows farmers to operate motor pumps for irrigation, enabling them to get extra income through the cultivation of vegetable gardens and thus to remain as cultivators. Private means of transportation, on the other hand, enable households to reach more marketing outlets to sell their agricultural products or buy agricultural inputs (confirming the results from Birthal et al. 2013; Levien 2013). Regarding community physical capital, the results show a negative effect of the proximity to markets and industrial areas on the odds of engaging in agricultural labour. The results also show that agricultural labourers who are located in communities with a greater community physical capital are more likely to be engaged in marginal employment. These two observations support the hypothesis that proximity to markets is associated with smaller farm holdings. Such farms do not require as much agricultural labour as other farms due to their small size, thus reducing the likelihood of agricultural labourers being hired throughout the year (Birthal et al. 2013; Levien 2013).

#### Human capital

The findings show that access to human household capital reduces the likelihood of engaging in agricultural labour for agricultural households, and reduces the likelihood of being employed marginally for agricultural labourers. Similarly, proximity to education and health facilities also reduce the likelihood of engaging in agricultural labour. A strong human capital enables households to be more resilient to climatic shocks by looking for temporary income-generating activities after facing an external shock and thus reducing the likelihood of selling their land and engaging in agricultural labour (Jansen et al. 2006). It also increases the availability of workforce during high demand periods of labour, such as crop establishment and harvest, during which all members work on the farm, reducing the need for extra labour costs. This corroborates previous findings, which showed that access to household human capital increases the chances of adopting mechanised commercial farming and to generate sustainable incomes (Paudel Khatiwada et al. 2017).

#### Financial capital

The results show that access to financial household capital reduces the likelihood of households engaging in agricultural labour. Access to financial services and the ownership of protective equipment (assets that can be sold if the household faces a shock) enable households to cope with crop failure and thus prevent them from selling their land after facing a shock. This corroborates previous findings which showed that access to household financial capital enables households to reduce the barriers to retaining a remunerative on-farm livelihood strategy such as cultivation (Babulo et al. 2008). Therefore, households that lack access to financial capital are more likely to sell their productive assets and to engage in agricultural labour. Land dispossession due to indebtedness was confirmed during the focus groups: households sell their land to cope with an external shock and become landless farmers. Interestingly, although participants flagged proximity to financial services as an important capital for their livelihood opportunities, our results show that agricultural households who benefit from greater access to community financial capital are more likely to be landless agricultural labourers. This rather counter-intuitive result is explained by the fact that proximity to financial institutions goes hand in hand with external investments that increase the pressure on farm holdings, thus encouraging smallholders’ land dispossession by larger farm holders (Birthal et al. 2013). Therefore, community financial capital indirectly increases the likelihood of being an agricultural labourer rather than a cultivator. The issue that emerges from these findings is that access to financial services (household financial capital) is a greater barrier to credit than access to financial infrastructures (community financial capital). Households may rely on the informal financial sector when they lack access to formal institutions, which traps them further into poverty.

#### Social capital

A low household social capital (weak kinship ties) is found to increase the likelihood to engage in daily-wage agricultural labour compared to cultivation for agricultural households, a result which mirrors the observations of Gang et al. (2008) who showed that socially excluded groups suffered from land market exclusion and a lack of employment opportunities. On the other hand, households with strong access to household social capital are less likely to be marginal agricultural labourers, thanks to their social networks that provide them with greater employment opportunities (Collier 2002). However, agricultural labourers who have access to greater community social capital are more likely to engage in marginal activities. The availability of recreational facilities (e.g. cinemas, stadiums, playgrounds) and of unions gives a greater possibility of kinship ties, which goes hand in hand with participation in such groups (Soltani et al. 2012) or enable households to move away from agricultural activities by providing them with off-farm livelihood alternatives. As mentioned during one focus group, this finding can be attributed to the time invested in such unions, especially Self-Help Groups, in order to develop income-generating activities for the future (Datta 2015). In such a case, a household’s strategy may be to keep a marginal labour activity to enable their members to get involved in the development of self-enterprise income-generating activities.

### Population density and agricultural labour

Rural population density has a major influence on the social and demographic aspects of rural communities, yet there are only a few analyses of their effects on agricultural labour employment (Smailes et al. 2002), most studies having looked at associations between population density and agricultural intensification (e.g. Josephson et al. 2014; Muyanga and Jayne 2014). The findings from this research show that agricultural households are less likely to be agricultural labourers in densely populated communities. This can be explained by the increased pressure on farm holdings in these areas, which encourages smallholders’ land dispossession by larger farm holders (Levien 2013). These newly landless agricultural households move out from agriculture and benefit from the economic opportunities that exist in highly dense areas to find off-farm livelihood alternatives (Muyanga and Jayne 2014). Another finding concerns agricultural households who live in the districts of Khordha and Jagatsinghpur: it appears that households from these districts have a greater likelihood to engage in agricultural labour. These results echo our qualitative findings, which demonstrated that there were high rates of emigration from these districts, partly due to the low incomes that cultivators receive from their farm and to the high proportion of agricultural labourers.

### Castes and agricultural labour

Although the caste system is no longer connected to the type of activities conducted by its members, high status employment is dominated by upper caste, while physical labour and low status jobs are mostly performed by lower caste or dalit (Levien 2015). Social and cultural norms in India limit people from the lowest caste to exercise their right to own and manage land and productive assets. As a consequence, landowners only rent land to farmers that are perceived as less risky, such as large farmers or farmers from the same socio-economic class and caste. Such a structure of land relations works as a barrier against scheduled castes and scheduled tribes’ economic agency and legal entitlements by preventing them from obtaining access to land (Kelkar and Kumar Jha 2016). By controlling for the proportion of scheduled castes and tribes, our findings show that belonging to disadvantaged castes is the underlying driver that explains the proportion of agricultural labour in a community.

### Policy relevance and suggestions for future work

The above findings suggest several courses of action for public policies and schemes in India to reduce rural outmigration and, thus, to reduce urban and rural poverty. The Mahatma Gandhi National Rural Employment Guarantee Act (MGNREGA) that guarantees 100 days of work at a fixed wage to rural dwellers seems to be well targeted to reduce the vulnerability of daily-wage agricultural labourers. However, important changes would need to be made to ensure that it plays a role in long-term poverty alleviation: although the scheme already works towards increased physical access to banks, there is a need to develop access to financial services as it decreases the likelihood for agricultural households engaging in agricultural labour. Moreover, it was shown that lack of access to financial services is a limit to the collection of MGNREGA wages as poorer households do not have access to bank services (Imai et al. 2010). The scheme should be used hand-in-hand with the National Rural Livelihood Mission (NRLM) to ensure work stability, especially during the lean season. Considering the wide implementation of Self-Help Groups in rural communities across all India (Datta 2015), embedding them better into policies would improve the provision of financial services to the most vulnerable households. Finally, agricultural tenancy laws should be implemented and enforced to regulate rents and offer security of tenure to tenants, as we demonstrated that larger farms lead to smallholders’ land dispossession and thus drive these households into agricultural labour. Interventions in property rights would prevent land grabbing by large farm holders (Sahu and Dash 2011) and would secure smallholders’ productive assets, thus reducing their likelihood to become agricultural labourers and fall into chronic poverty.

This research makes several contributions to the body of literature on livelihood studies. The current findings show the importance of separating community resources from household capitals to characterise decisions about rural livelihoods. This approach defined a set of indicators that adequately capture the multidimensional and multi-attribute nature of rural communities and household capitals. Two different methods were used to obtain the final results: a deductive binning of indicators into different categories based on rapid rural appraisals, followed by an inductive indicator method constructed via principal components analysis for community and household capitals. Overall, identifying community capitals is useful for assessing needs and targeting intervention or mitigation programmes. It provides an approach for practitioners and policy-makers to take into account the contextual factors that drive livelihood precarity and thus to target more strategically anti-poverty programmes or activities to maximise their effect rather than equally distributing them across all places. For example, interventions should focus on strengthening human and physical capitals in communities with a low natural capital to ensure that households are able to diversify their livelihoods to off-farm strategies, while they should be targeted on providing financial capital and complementary livelihood opportunities during the lean season in communities with low financial and physical capital.

## Conclusion

The present study sought to determine the influence of community capitals and household capitals on agricultural employment. Our findings bring a new perspective on the determinants of rural poverty by demonstrating that both community and household capitals have an influence on agricultural livelihood opportunities. This study also shows that community resources and household capitals should be considered separately as they do not necessarily have the same effects on the likelihood of being a landless agricultural labourer. Our approach using multilevel modelling is an appropriate framework to support this differentiation.

Our results show that human, financial and social household capitals reduce the likelihood of engaging in daily-wage labour for agricultural households. Our findings suggest that households are more likely to be landless agricultural labourers near well-connected rural centres, due to smallholders’ land dispossession by larger farm holders and dynamics of in-migration. Another important result is that agricultural labourers are more likely to have marginal employment in remote areas, which makes them amongst the poorest socio-economical group in rural India. These findings suggest that investment in rural infrastructure might increase livelihood vulnerability, if not accompanied by an improvement in the provisioning of complementary rural services, such as access to rural finance, and by the implementation of agricultural tenancy laws to protect smallholders’ productive assets.

## Electronic supplementary material

Below is the link to the electronic supplementary material.

Electronic supplementary material 1 (PDF 183 kb)

## Footnotes

1 The products were retrieved from the Bhuvan website, courtesy of the National Remote Sensing Centre (NRSO), Indian Space Research Organisation, http://bhuvan.nrsc.gov.in.
