# Supplementary material for: Collective influence of household and community capitals on agricultural employment as a measure of rural poverty in the Mahanadi Delta, India
Source: Ambio. 2019 Mar 9;49(1):281–98. doi: 10.1007/s13280-019-01150-9 (PMC6889257; doi:10.1007/s13280-019-01150-9)
Supplement: Supplementary file 1 — Electronic supplementary material 1 (PDF 183 kb) [file 13280_2019_1150_MOESM1_ESM.pdf]

Ambio

Electronic Supplementary Material
This supplementary material has not been peer reviewed.

Title: **Collective influence of household and community capitals on agricultural employment as a measure of rural poverty in the Mahanadi Delta, India**

Authors: Tristan Berchoux, Gary R. Watmough, Fiifi Amoako Johnson, Craig W. Hutton, Peter M. Atkinson

S1 - Results from the Rapid Rural Appraisals

Local residents and stakeholders identified key factors which impact their livelihood opportunities. These were grouped into different capitals based on the SLF. Private assets were classified as household livelihood capitals whilst public and common-pool assets were classified as community capitals. The capitals were further categorised into natural, physical, human, financial and social capitals based upon the views of the stakeholders and local residents. The following section provides a reminder of the main capitals highlighted and their associations with livelihood activities. It provides a necessary summary of the context and background for the statistical analysis as to which capitals are determinant for livelihood opportunities.

S1.1. Livelihood capitals

Natural capital. According to the participants, there were three main components that fell into natural capital. These were, by order of importance: (i) agricultural land, which was mentioned as both community and household capitals; (ii) access to fresh water, which was considered as a community capital; and (iii) access to forest resources, considered as a community capital.

The most important component raised by the participants was land, used for agricultural purposes. Different characteristics fell under this natural capital component: (i) the size of land available to one household (household capital); (ii) the ownership status of this land (household capital), which is shaped by social relations of class and caste; (iii) the size of cropland, pasture and fallow available in the community (community capital); and (iv) the topography and agro-meteorology of the land (community capital). The number of acres available per household was, according to the participants, the capital with the greatest influence on households' choice of a livelihood strategy. Indeed, households who had access to a greater number of acres (two acres and above) produced enough to be food secure during the whole year. The rest of the production could be sold or the extra land could be used for commercial crops such as cashewnuts cultivation, betel (leaf used in paan for chewing) or coconut plantation. On the contrary, households with little access to agricultural land (less than one acre) could not produce enough food to be food secure, hence they had to go for extra income-generating activities to reach food security. Land ownership was also raised as a determining capital for livelihood strategies: share-croppers had to give a part of their production to the land owner (around 50% of the harvest), who are usually from higher castes. As a consequence, a share-cropper with access to one acre of land had, in fact, a production of only half acre of land (despite working on one acre of land).

> "A sharecropper with 1 acre of land has to give half of the harvest to the owner, who is from outside the village most of the time." - *Male participant, community C8* -

The total area of agricultural land (including cropland, pasture and fallow) was perceived as a positive community asset, especially in remote communities. According to participants, a greater area of cultivated area in the village enabled the creation of a supply force that would attract traders to come. For example, in the community C10, it was the fact that many households decided to breed goats that led traders to come and buy them. Interestingly, households who were engaged in non-agricultural activities also confirmed that a greater total surface of agricultural land in the village catalyses economic activities and livelihood opportunities. For agricultural households, the topography of the village was perceived as a key resource or as a key problem depending on the community visited (Table 1): communities who only had access to high lands for agriculture could cultivate their crops during one season only (*kharif*) and had

to leave the land barren during *rabi*, while the one with access to low land could cultivate two crops per year (with an associated increase of flood risk).

Table 1: Main types of land in the Mahanadi Delta based on topography. As defined by Odisha's department of agriculture and farmers' empowerment (source: authors' interviews).

**Land** *(type)*	**Cultivated area** *(10^5 hectares)*	**Paddy** *(%)*	**Characteristics**
High	29.14	36	Drought-prone, no irrigation, low yield, usually one season
Medium	17.55	91	Flood-prone (flash floods), lower yield than low land
Low	14.82	98	Flood-prone (water logging), irrigated, high paddy yield

Beside land, communities raised the importance of access to open-water resources and to forest resources (community capitals). Proximity to a lake, a river or the sea gave households the opportunity to diversify their livelihood activities and food security through fishing, shrimp farming or kitchen gardening (manual irrigation from local ponds). Concerning forest resources, different products were traded, such as timber (wood, charcoal) and non-timber forest products (bamboo, *sal* seeds, *kendu* leaves and *mahuwa* flowers).

Physical capital. At the community level, road connectivity and proximity to marketing outlets appeared to be the most important assets. At the household level, access to productive assets (for agriculture, fisheries, or handicraft) and to means of transportation were the most important assets raised by participants.

Having access to all-weather transportation infrastructures (roads) was perceived as a factor that improves working opportunities through access to marketing outlets (traders are able to come to buy goods directly in the community). Although households benefit differently from such assets depending on their wealth and social networks, proximity to a marketing outlet and availability of electricity in the village were mentioned as key determinants to develop income-generating activities. A marketing outlet could be of different types, from general (such as a market) to more specific (such as a cooperative or society), proximity to an outlet acting as a catalyst for activity diversification, such as milk or raw-fish production.

> "After the creation of the milk society 7 years ago, we started to breed Jersey cows because they give more milk. Now we sell our milk there everyday, and it is it located at a walking-distance." - *Male participant, community C4* -

The availability of irrigation canals and tanks in the community, mainly associated with the green revolution, plays a major role into mitigating the effects of climate variability on agricultural production (through droughts or floods) and was mentioned as a key community capital. Such irrigation facilities are considered as common-pool resources because they were publicly funded by the State Surface Flow Irrigation Schemes and most households are able to benefit from them regardless of their class or castes. For example, participants from non-agricultural households mentioned that they were able to collect water from the canals to irrigate their house gardens for their supply of vegetables.

Access to productive assets, such as draft animals, equipment (seeds, fertilisers), machinery (tractor with plow, water-pump, fishing boats) and means of transportation (bicycle, motorcycle, car), was also a raised by communities as determinants for their livelihoods. Means of transportation (either private or public) allowed households to look for new marketing outlets

for their production and also to reduce travel times to nearby services. For the participants, investing in productive assets would enable them to increase their agricultural or fishing productivity, thus increase incomes (for the same workforce and time spent). For example, some households were able to cultivate during *rabi* thanks to irrigation systems they had invested in, such as water pumps.

> "We can't cultivate during *rabi*, there is water scarcity [...] I am the only one in the community who cultivates during *rabi* season, thanks to the water-pump I bought. I have 3 acres and I produce pulses, ginger, cucumber, sunflower and watermelon." - *Female participant, community C9* -

Human capital. According to the sampled communities, workforce (considered as a household capital), education (considered either as a household or community capitals) and sanitation facilities (considered as a community capital) were perceived as the most important assets to take into account in the human capital.

Due to the gendered division of labour, male participants described male workforce availability as the most important component of human capital for livelihood opportunities. Men were found to be in charge of cultivation and of earning incomes (through migration or daily-wage employment). For example, a household with only one man tended to engage in agriculture and would not be able to migrate as he had to look after the farm. As a consequence the household would look for daily-wage labour to diversify their incomes. On the contrary, if the household had more than 2 men, at least 1 man stayed to look after the farm and the rest were going for migration for 6 months (off-*kharif* season). The overall number of active members in the household also had a great influence on the range of livelihood activities the household could put in place. A great number of members allowed the household to cope faster in the case of an external shock, as all members could look for income-generating activities (mainly daily-wage labour). However, it was perceived that "large households" was a negative asset, as it was creating extra expenses for the households and increased the risk of food insecurity (decreased cropland area per person and thus problems of food security). It is interesting to note that women did not mention male workforce as a capital but they raised it as a social constraint that prevented the household to diversify their activities.

> "We didn't inherit from any land, we do share-cropping. We have three daughters, so my husband is the only man in the household. He cannot go for migration, he has to stay to take care of the household and of our agricultural land. So he is doing daily wage labour and we also rely on cow milk production." - *Female participant, community C4* -

At the household level, the presence of educated and skilled members was perceived as driving households towards a diversification of their activities. Actually, educated members were more likely to get a permanent employment in the public sector, such as teacher or administrator (at the *panchayat* or block level), or in the private sector (hairdresser, driver, etc.). They also were more likely to set up their own business such as service provider or trader. In some cases, there were also some specific skills that enabled members to go for "skilled" migration. The most famous example raised during the rapid rural appraisals were the plumbers from Pattamundai in Kendrapara, commonly called the "Plumbing Capital of India", who were going on long-term migration in other States of India and abroad and who were sending remittances back to their household in the community.

Finally, one of the main issues raised by the women was their lack of access to community amenities, such as schools, sanitation facilities (latrines, drinking water) and to health facilities (health centre, hospital). According to them, a better access to health facilities and to safe water

infrastructures would diminish the risk of health problems, while access to schools would enable their children to spend their day there, giving them time for other activities and increasing their future livelihood opportunities. Overall, they argued that proximity to these community amenities would enhance their labour capacity.

Financial capital. According to the participants, the main financial resources of one household are invested in protective assets such as electronics (TVs, radios, phones), furniture, clothing and jewellery. For household who were not involved in livestock rearing, ownership of cattle and goat was also considered as a protective asset, used as a saving and insurance instrument. Protective assets were to be sold if the household faced an external shock (crop failure, death, disease, wedding). Having access to financial services was mentioned by the participants as providing two different services, savings and loans, although only better-off households were able to have savings in a bank account. Access to loans allowed households to invest in their means of production (agriculture or other income-generating activities) or to cope with external shocks and reduced the likelihood of distress migration.

Participants primarily mentioned the importance of having access to banks in order to obtain formal financial services. However, due to the privatisation of these institutions after the liberalisation, tenant cultivators are denied access to formal credits. The inability for smallholder farmers to access formal credit forces them to rely on "informal sources", such as the traders who provide agricultural inputs or local moneylenders with usurious interest rates. Such a dynamics pushes farmers into a long-term indebtedness, which undermine their future financial capital and livelihoods. At the community-level, proximity to a bank was also raised as critical when it comes to state schemes and pensions: for example households needed a bank account in order to get paid for work they conducted under the Mahatma Gandhi National Rural Employment Guarantee Act (MGNREGA). Female participants emphasised the importance of Self-Help Groups as alternatives to traditional financial services as they argued that they could get access to loans through them.

Social capital. Participants mentioned the importance of social groups such as Self-Help Groups (SHG), youth and farmers groups to give them new income opportunities or to increase their migration options. These groups are considered as community-level assets, which enhance social networking that might lead to alternative livelihood opportunities. Participants, and especially women, showed a strong interest in SHGs, which is a way for them to build strong social links and to build their capacities and empower themselves. It also emerged from the discussion that availability of recreation facilities, such as *chaupal* (public community space or building) or sport fields was an important community capital that enabled to build strong kinships and that also prevented younger males to migrate out of the village for work.

Some participants felt that family connections were a key asset to find job opportunities and to be integrated into migration networks, arguing that household size helps to expand networks. While discussing with widows, it emerged that unmarried or widowed households were to suffer from social exclusion, especially from community groups and unions. Interestingly, no participants mentioned the issues of scheduled castes and tribes until a conflict emerged during one of the activity. Separate discussions were then held with the participants involved in the incident and the theme of social balance emerged. Participants from a caste in minority in the community reported that the unbalanced ratio between general castes and scheduled castes, tribes and other backward castes had led to one caste taking over the other and to the exclusion of some of them from the community social groups such as SHG. As a consequence, participants felt that their community networks were impoverished.

> "In our hamlet, scheduled caste is the main population; we general caste are a minority now. SC are the majority and they have a strong voice so they have the power. As general caste, we do not benefit from governmental schemes and subsidy loans for SHG, whatever is left, we get that. So the SC women asked us to leave SHG groups, there are no more mixed SHG groups now. We keep silence to keep no tension, but if we want to raise tension, then there will be tension." - *Female participant, community C1* -

S1.2. Local perceptions of the effects of livelihood capitals on activities

The findings from rapid rural appraisals show that participants perceive that there is a link between households' access to livelihood capitals and their choice of a livelihood activity.

Household-level drivers. On-farm activities as main livelihood are driven by a great access to natural and physical capitals. Large farms provide higher incomes to farmers and therefore, increase farm survival, as suggested by Kimhi and Bollman (1999). Quality and quantity of agricultural land have a positive effect on engaging into cultivation, while having access to productive capital is likely to lead to a specialisation into commercial agriculture. The caste system also plays a role in the choice of conducting an activity, for example fishing: fishermen is perceived as a job for the low castes. As a consequence, dwellers from higher castes do not go fishing, even if they have access to water resources and even under the circumstance of an external shock.

> "We can't go fishing because we are not from the fisherman caste, we are from the general caste." - *Male participant, community C3* -

The drivers of off-farm activities mainly fall under human, financial and social capitals. Permanent employment and self-employment are both positively influenced by the level of education of the household's members and by their ownership of protective assets. Starting a business or migrating requires a financial input, either to buy equipment to start the business, or to pay for transport and accommodation for migrants. Moreover, being able to migrate also depends on the strength of social networks (or migration networks) and on the communication facilities one household has access to. Households take the decision to have one of their members migrating only if there is a man that can stay to take care of the farm. Availability of male workforce is a key driver of migration and more specifically of seasonal migration.

Community-level drivers. According to the findings, livelihoods were found to be shaped by their geography and access to common goods, managed at the community level. The literature points out that access to common-pool resources is shaped by social relations of castes, lowest castes being prevented to access water or forest resources (e.g. Borooah et al. 2014). However, the rapid rural appraisals conducted within this study showed that access to norms of self-identity, with middle-castes preventing themselves to use some common-pool resources, as it might be seen as an activity for lower castes, confirming previous studies (e.g. Sankaran et al. 2017). Similarly, regarding irrigation facilities, although clandestine encroachment and tampering with the water course can be found among wealthy farmers of dominant castes, who rely upon their status to assuage dissent and on political connections to suppress official complaints, inequalities in water access depend more on the ability to monopolise groundwater supplies by digging expensive and uncertain bore-wells than on monopolisation of tank water.

The total agricultural area of the community was perceived as a stimulating factor for cultivation, as it motivates traders to come buy the goods directly in the community. Evidence

suggests that an increase in the access to operational land reduces the tendency to close down farms, thus reducing the likelihood of farm exit and of households engaging in precarious livelihoods (Glauben et al. 2006; Bhandari 2013). The total agricultural area in the village has a positive effect over the possibilities of other capitals: it can create synergies between farmers to buy agricultural equipment (physical capital), invest into irrigation infrastructures (physical capital) or in can increase their bargaining power (Courtois and Subervie 2015; Agarwal 2018). The success of most agricultural activities depends on the capacity of households to sell their products and so is also dependent of a good road connection with an outlet nearby. Access to water resources is a *sine qua non* condition to conduct fishing activities, but making a living out of it also requires an access to outlets to sell the products and to private fishing equipment. Concerning forest resources, activities are independent of the existence of an outlet nearby, they rely on good road connections and on the area of forest available, which provides households with high value-added products (*sal* seeds, *kendu* leaves, *mahuwa* flower). Selling these products to traders that come directly to the communities to buy the goods enable households to earn extra income and to cope better with external shocks. Access to communal lay land, defined as customary communal tenure that can be used for animal grazing, is an incentive to put in place livestock rearing activities.

The main difference between the two communities relies upon the proportion of dwellers involved in "others" activities, which can be attributed to their road connectivity, *Keutajanga* benefiting from the proximity to a trading-centre (Puri), while *Kusupalla* is more remote. Proximity to trading-centres with community amenities was perceived as driving off-farm activities. This can be explained by the very good connectivity and the proximity of a market for both communities. It is interesting to note that *Dakhinaveda* and *Loknathprasad* have a very different structure of livelihood activities even though they are located nearby, thus should benefit from a similar access to natural and physical capitals. A possible explanation for this might be that both communities suffer from land erosion, Loknathprasad being much more affected due to its exposure to three rivers whereas Dakhinaveda is exposed to one. As a conclusion, participants perceived that their access to community capitals have an influence on the type of livelihood activities they put in place. Natural and physical community capitals are perceived as drivers of on-farm activities, while the combination of social, financial and human community capitals seem to induce off-farm activities.

S2 - Principal Component Analysis

S2.1. Measuring household capitals

Private assets were grouped together and classified as household livelihood capitals.

Measuring household natural capital. A common view amongst participants was that the amount of agricultural land (rainfed and irrigated cropland, tree plantation) available to one household influences their potential income and food, and they considered them as determining factors for their choice of a livelihood activity. Participants in inland communities (C2, C5 and C6) argued that the area of pasture available per household was also a key determinant of employment, as it enabled them to develop livestock rearing as a diversification strategy. The four highest loadings of the eigenvector from the Principal Component Analysis represent these capitals highlighted by participants as determinants for the choice of their livelihood strategy: cropland area per cultivator ($\lambda_{\text{cropland}} = 0.38$), area of pasture per household ($\lambda_{\text{pasture}} = 0.44$) and area of tree plantation per cultivator ($\lambda_{\text{tree plantation}} = 0.40$).

Measuring household physical capital. A number of factors falling under household physical capital were identified by participants as determinant in their choice of a livelihood strategy. First, private access to electricity enables households to conduct their livelihood activity by operating agricultural pumps and machinery ($\lambda_{\text{no\_electricity}} = -0.08$). Means of transportation ($\lambda_{\text{bicycle}} = 0.45$, $\lambda_{\text{motorcycle}} = 0.53$, $\lambda_{\text{car}} = 0.40$) also came up during the rapid rural appraisals, since they allow households to look for new outlets for their production or for livelihood opportunities and increase their access to nearby services (hospitals, banks, schools) through the reduction of travel times.

Measuring household human capital. A recurrent household human capital that was identified by participants as influencing their choice of a livelihood strategy was the number of active members in the household ($\lambda_{\text{dependencyratio}} = -0.69$). A high dependency ratio limits the range of activities that one household can put in place. Finally, level of education and individual skillsets surfaced in most focus groups. Participants argued that educated members were a strength for one household because they "did not suffer from unemployment". Based on existing literature about poverty, levels of female illiteracy were used as a negative proxy for this asset ($\lambda_{\text{illiteracy}} = -0.69$).

Measuring household financial capital. One of the proxies used to quantify household financial capital are households' access to financial services for savings and credits ($\lambda_{\text{financial\_services}} = 0.68$). This indicator only captures financial inclusion as defined in the census: only households with access to banking services provided by nationalised banks, private banks, foreign banks and co-operative banks are considered to have access to financial services. However, many smallholder farmers –particularly households from lower castes and the poor– lack access to formal credit and are forced to rely on semi-formal (credit and thrift societies, self-help groups, primary agricultural credit societies) or informal (moneylenders and shopkeepers) sources. Moreover, access to such financial services can become a negative asset when the debt-to-capital ratio is greater than one. Participants also identified housing as a measure of the financial capital available to one household, as it is associated with access to financial services. Based on census variables, housing condition was used as a proxy to represent such an asset ($\lambda_{\text{dilapidated}} = -0.68$).

Measuring household social capital. Household social capital is about the value of social networks, including bonding with norms of reciprocity. Although not identified clearly as a capital, it emerged from the focus groups that marriage is one of the most important kinship encountered at the household level in rural settings, and so one of the pillar of social capital. Households' marital status was used to represent such kinships ($\lambda_{\text{married\_0}} = -0.40$). Finally, participants mentioned that households who owned a mobile phone had stronger social networks, especially outside the village, enabling them to have access to alternative livelihood opportunities ($\lambda_{\text{telephone}} = 0.57$).

S2.2. Measuring community capitals

After reviewing the determinants of households' livelihood strategies identified by the participants, public and common-pool assets were grouped together and classified as community livelihood capitals.

Measuring community natural capital. Participants, in particular those from remote communities, argued that the total amount of land in the community was a driver of agricultural livelihoods, as it would increase opportunities for agricultural labour and agricultural marketing. A greater area of cultivated area in the village enables the creation of a supply force that can attract traders to come, as it was the case in the community C10 where an increase in the number of breeding goats households had attracted traders to come, thus creating new livelihood opportunities, such as goat broker. Households who were engaged in non-agricultural activities also argued that the greater the total surface of agricultural land in the village, the more economic activities and livelihood opportunities there are. As a consequence, the area of potential cropland was considered as a positive community capital and included in the quantification of its indicator ($\lambda_{\text{crops}} = 0.65$). Forest resources were unanimously raised by participants as a common-pool capital in communities located near forests. Different products from the forest can be traded, such as timber (wood, charcoal) and non-timber forest products (bamboo, sal seeds, kendu leaves and mahuwa flowers), enabling households to diversify their incomes. As the availability of products in a forest is correlated with its size, this resource was proxied by the total area of forest accessible to the village, computed from satellite imagery ($\lambda_{\text{forest}} = 0.20$). Similarly, proximity to water resources or community ponds was unanimously flagged by participants as a source for alternative livelihoods and extra incomes through aquaculture ($\lambda_{\text{openwater}} = -0.59$). Finally, the availability of irrigation infrastructures in the community was mentioned as a determinant public good that enabled them to increase their income ($\lambda_{\text{irrigation}} = 0.34$).

Measuring community physical capital. The importance of community physical capital to influence the choice of a livelihood strategy recurred throughout the focus groups. Although households benefit differently from such assets depending on their wealth and social networks, proximity to a marketing outlet and availability was mentioned as key determinants to develop income-generating activities ($\lambda_{\text{markets}} = -0.53$). A marketing outlet could be of different types, from general (such as a market) to more specific (such as a cooperative or society), proximity to an outlet acting as a catalyst for activity diversification, such as milk or raw-fish production. Finally, the availability of industrial areas near the community ($\lambda_{\text{industry}} = -0.53$) is a positive community asset increasing households' employment opportunities.

Measuring community human capital. A number of themes falling under community human capital emerged from the focus groups. Participants argued that proximity to medical, educational and water community amenities would enhance their labour capacity. Availability of education in the premise of the community recurred throughout the discussions, especially during focus groups held with women. They argued that access to schools would enable their children to spend their day there, giving them time for other activities and increasing their future livelihood opportunities. Education scores were computed from the census using Euclidian distance to nearest secondary school ($\lambda_{\text{school}} = -0.70$). Another recurrent theme was the issue of distance to health facilities ($\lambda_{\text{medical}} = -0.70$). According to them, a better access to health facilities and to safe water infrastructures would diminish the risk of health problems.

Measuring community financial capital. Proximity to a bank was raised as critical when it comes to state schemes and pensions: for example households needed a bank account in order to get paid for work they conducted under the Mahatma Gandhi National Rural Employment Guarantee Act (MGNREGA). As a consequence, infrastructures linked to formal financial services were included, such as the distance to the nearest commercial bank ($\lambda_{\text{bank}} = -0.58$) and ATMs ($\lambda_{\text{ATM}} = -0.41$). Two other types of infrastructures specific to the Odisha context were flagged by participants: the distance to Public Distribution System Shops ($\lambda_{\text{PDS}} = -0.69$), which are

shops distributing rations at a subsidised price to the poor. Although PDS shops are not financial institutions, it was decided to include them in the community financial capital because they are used as a policy financial tool for poverty reduction.

Measuring community social capital. Community social capital emerged discretely from the focus groups, as the concept of social networks at a community level was not identified by participants. However, participants mentioned the importance of social groups such as self-help groups ($\lambda_{\text{SHG}} = -0.32$), youth and farmers groups to give them new income opportunities or to increase their migration options. These groups are considered as community-level assets, which enhance social networking that might lead to alternative livelihood opportunities. Participants, and especially women, showed a strong interest in SHGs, which is a way for them to build strong social links and to build their capacities and empower themselves. It also emerged from the discussion that availability of recreation facilities, such as public community spaces ($\lambda_{\text{community centre}} = -0.34$) or sport fields ($\lambda_{\text{sport field}} = -0.68$) was an important community capital that enabled to build strong kinships and that also prevented younger males to migrate out of the village for work.

References

1. Agarwal B (2018) Can group farms outperform individual family farms? Empirical insights from India. World Development 108:57–73, DOI 10.1016/j.worlddev.2018.03.010, URL http://linkinghub.elsevier.com/retrieve/pii/S0305750X18300913
2. Bhandari PB (2013) Rural livelihood change? Household capital, community resources and livelihood transition. Journal of rural studies 32:126–136, DOI 10.1016/j.jrurstud.2013.05.001, URL http://www.pubmedcentral.nih.gov/articlerender.fcgi?artid=3772533{&}tool=pmcentrez{&}rendertype=abstract
3. Borooah VK, Diwakar D, Mishra VK, Naik AK, Sabharwal NS (2014) Caste, inequality, and poverty in India: A re-assessment. Development Studies Research 1(1):279–294, DOI 10.1080/21665095.2014.967877, URL http://dx.doi.org/10.1080/21665095.2014.967877
4. Courtois P, Subervie J (2015) Farmer Bargaining Power and Market Information Services. American Journal of Agricultural Economics 97(3):953–977, URL http://dx.doi.org/10.1093/ajae/aau051
5. Glauben T, Tietje H, Weiss C (2006) Agriculture on the move: Exploring regional differences in farm exit rates in Western Germany. Jahrbuch fur Regionalwissenschaft 26(1):103–118, DOI 10.1007/s10037-004-0062-1
6. Kimhi A, Bollman R (1999) Family farm dynamics in Canada and Israel: The case of farm exits. Agricultural Economics 21(1):69–79, DOI 10.1016/S0169-5150(99)00015-8
7. Sankaran S, Sekerdej M, von Hecker U (2017) The role of Indian caste identity and caste inconsistent norms on status representation. Frontiers in Psychology 8(MAR):1–14, DOI 10.3389/fpsyg.2017.00487
